# Epidemiological trends of female breast and gynecologic cancers in adolescents and young adults in China from 1990 to 2019: Results from the Global Burden of Disease Study 2019

**DOI:** 10.3389/fonc.2022.1003710

**Published:** 2022-10-13

**Authors:** Yuting Zhao, Pei Qiu, Lutong Yan, Shouyu Li, Zejian Yang, Huimin Zhang, Jianjun He, Can Zhou

**Affiliations:** ^1^ Department of Breast Surgery, The First Affiliated Hospital of Xi’an Jiaotong University, Xi’an, China; ^2^ School of Medicine, Xi’an Jiaotong University, Xi’an, China

**Keywords:** breast cancer, gynecologic cancer, global burden of disease, incidence, death, disability-adjusted life years

## Abstract

**Background:**

Research on the incidence, mortality, and disability-adjusted life years (DALYs) of female breast and gynecologic cancers (FeBGCs) and the relevant risk factors for adolescents and young adults (AYAs) are valuable for policy-making in China. We aimed to estimate the incidence, deaths, and DALYs and predict epidemiological trends of FeBGCs among AYAs in China between 1990 and 2019.

**Methods:**

Data from the 2019 Global Burden of Disease (GBD) study between 1990 and 2019 in 195 countries and territories were retrieved. Data about the number of FeBGC incident cases, deaths, DALYs, age-standardized rates (ASRs), and estimated annual percentage changes (EAPCs) were extracted. A comparative risk assessment framework was performed to estimate the risk factors attributable to breast cancer deaths and DALYs, and autoregressive integrated moving average (ARIMA) models were fitted for time-series analysis to predict female cancer morbidity and mortality among Chinese AYAs until 2030.

**Results:**

In 2019, there are 61,038 incidence cases, 8,944 deaths, and 529,380 DALYs of FeBGCs among the AYAs in China, respectively. The estimated annual percentage change (EAPC) values were positive scores (>0) in ASIRs and negative scores (<0) in ASMR and ASDR. Furthermore, in 2030, the incidence rate of FeBGCs would grow to 30.49 per 100,000 in China, while the mortality rate would maintain a steady state. Of the deaths and DALYs, diet high in red meat was the greatest contributor to breast cancer, while a high body mass index (BMI) was the greatest contributor to cervical, ovarian, and uterine cancers.

**Conclusion:**

The increasing Chinese FeBGC burden is mainly observed in AYAs and non-red meat diet, and the control of body weight could reduce FeBGC burden in China.

## Introduction

Female breast and gynecologic cancers (FeBGCs) are the most frequent health issues and the leading cause of cancer-related deaths in women (1, 2), especially female adolescents and young adults (AYAs) (3). Due to the rapid socioeconomic transition and opening up to the outside world over the past three decades, different social norms and values related to sex and marriage have been exposed to AYAs. This rapid societal change also contributed to the development of diagnostic activities linked to the screening programs for breast and cervical cancers and increasingly urbanized and westernized lifestyles, particularly the growth in the prevalence of obesity and physical inactivity. Moreover, the incidence of FeBGCs has escalated in China over the past three decades (4, 5). What is even more concerning, FeBGCs are characterized by the correlation between the factors of reproduction, hormones, behavior (6–9), functional lesion infertility, and sexual and mental health of women (10–12).

Meanwhile, cancers in women of childbearing age tend to show different characteristics and prognoses when compared with those in elderly patients (13–15). In China, the female AYAs have a large percentage of 16.65% of the Chinese population with an estimated 114 million of its population in the 15–39-year age group in 2019 (16). It is remarkable that female AYAs have a higher demand for fertility, sex, and body image, although function in fertility, the image of the body, and sex could be damaged during the diagnosis and treatment of FeBGCs (12). Therefore, an in-depth study should be carried out to illustrate the profile and risk factors attributable to the disease of FeBGCs, reduce the burden of the disease, and promote mental and physical health for women in China. However, until now, there has been no scientific evidence to estimate the incidence, deaths, and DALY and predict epidemiological trends of FeBGCs among AYAs in China.

This study sought to describe the trends of incidence, mortality, DALYs, and risk factors of FeBGCs among the AYAs in China from 1990 to 2019 and predict the incidence and mortality by 2030. To our knowledge, this is the first study to focus on Chinese AYAs with female cancers and to predict morbidity and mortality using time-series analysis. In addition, to seek the gap between China and the world, especially the developed countries, we also analyzed the data of the globe, the United States of America (USA), and five social-demographic index (SDI) regions. The findings of the current research could provide etiological clues for the prevention of FeBGCs in Chinese AYAs, illustrate women’s health promotion and health resource allocation in the future, and provide a reference for other developing countries.

## Methods

### Data sources

Data for this study are derived from the Global Burden of Disease (GBD) 2019 study, which assesses the comprehensive epidemiological indicators of the burden of 369 diseases and injuries, including the morbidity, prevalence, mortality, years of life lost (YLLs), years lived with disability (YLDs), and disability-adjusted life-years (DALYs) due to the 369 diseases and injuries, for 204 countries and territories from 1990 to 2019, with two genders included. The general methodology for estimating causes of death and disease incidence and prevalence has been clarified in previous publications (1, 17, 18).

We searched and collected the incident cases, mortality, DALYs, and risk factors of breast, cervical, ovarian, and uterine cancers of women diagnosed between the ages of 15 and 39 years in the globe, China, the USA, and five SDI regions. SDI is a compound measure of income, average years of schooling, and fertility for each GBD location and year which is used as a measure of socio-demographic development. The GBD 2019 study divided the SDI into quintiles from 0 to 1; thus, all GBD locations were divided into five categories: high, high-medium, medium, low-medium, and low SDI regions. Data used in the current study could be downloaded from the GBD Results Tool provided by the Global Health Data Exchange (GHDx) data catalog, which was created and supported by the Institute for Health Metrics and Evaluation (IHME) (19).

### Definitions

The AYAs referred to the age range from 15 to 39 years (3, 20). FeBGCs referred in this study included breast cancer (International Classification of Disease for Oncology, third edition [ICD-O-3] code: C50, C50.0, C50.01, C50.011, C50.012, C50.019, C50.02, C50.021, C50.022, C50.029, C50.1, C50.11, C50.111, C50.112, C50.119, C50.12, C50.121, C50.122, C50.129, C50.2, C50.21, C50.211, C50.212, C50.219, C50.22, C50.221, C50.222, C50.229, C50.3, C50.31, C50.311, C50.312, C50.319, C50.32, C50.321, C50.322, C50.329, C50.4, C50.41, C50.411, C50.412, C50.419, C50.42, C50.421, C50.422, C50.429, C50.5, C50.51, C50.511, C50.512, C50.519, C50.52, C50.521, C50.522, C50.529, C50.6, C50.61, C50.611, C50.612, C50.619, C50.62, C50.621, C50.622, C50.629, C50.7, C50.8, C50.81, C50.811, C50.812, C50.819, C50.82, C50.821, C50.822, C50.829, C50.9, C50.91, C50.911, C50.912, C50.919, C50.92, C50.921, C50.922, C50.929), cervical cancer (ICD-O-3 code: C53, C53.0, C53.1, C53.3, C53.4,C53.8, C53.9), ovarian cancer (ICD-O-3 code: C56, C56.0, C56.1, C56.2, C56.4, C56.9) and uterine cancer (ICD-O-3 code: C54, C54.0, C54.1, C54.2, C54.3,C54.4, C54.8, C54.9) provided in the GBD 2019 study.

YLDs means the years lived with any short-term or long-term health loss weighted for severity by the disability weights. YLLs means years of life lost due to premature mortality. DALYs refers to the sum of years lost due to premature death (YLLs) and years lived with disability (YLDs), also defined as years of healthy life lost. The estimation process has been fully described in previous publications (2, 17). Certainty of the estimation is represented as uncertainty interval (UI), for every estimate in GBD is calculated 1,000 times, with each time sampling from distributions for data inputs, data transformations, and model choice. The 95th uncertainty interval is determined by the 25th and 975th values of the 1,000 values after ordering them from smallest to largest.

### Statistical analysis

Age-standardized rates (ASRs), including age-standardized incidence rate (ASIR), age-standardized mortality rate (ASMR), and age-standardized DALY rate (ASDR), were calculated using the direct method with the World Health Organization’s (WHO) world standard population (2000–2025) based on the following formula:


$$\text{ASR}=\;\frac{\sum_{i=1}^{\text{A}}{\text{а}}_{i}{\text{w}}_{i}}{\sum_{i=1}^{\text{A}}{\text{w}}_{i}}\times 100,000$$

A linear relationship was hypothesized between the natural logarithm of ASR and time so that the estimated annual percentage changes (EAPCs) of ASRs with confidence intervals (CIs) could be calculated to describe the ASR change trends from 1990 to 2019 based on the following formula:


EAPC= 100 ×(exp(β)-1

If EAPC and the lower limits of CI were positive, the ASR tended to increase. On the contrary, if EAPC and the upper limits were negative, the ASR tended to decrease.

Autoregressive integrated moving average (ARIMA) models were fitted for time-series analysis to predict female cancer morbidity and mortality among the Chinese AYAs until 2030. The process of model construction included 1) stationary test, 2) model identification, 3) parameter estimation, 4) model diagnosis, and 5) model prediction. The correlation between EAPC and ASR was analyzed by Pearson’s relative correlation. Two-sided *P*< 0.05 was considered to have statistical significance. Statistical analyses were performed by GraphPad Prism version 7.00 and software package R version 4.1.3.

This study was approved by the Ethical Committee of the First Affiliated Hospital of Xi’an Jiaotong University. The GBD data erase the identity information of patients, so there is no need for informed consent from the patients included.

## Results

### The FeBGCs’ incidence, mortality, and DALY burden on the AYAs

Globally, there are approximately 343,281 (95% UI = 298,964–383,584) incident cases of female breast and gynecological cancers (FeBGCs), 80,614 (95% UI = 70,496–90,189) deaths, and 4.7 (95% UI = 4.1–5.2) million DALYs among the AYAs, and 61,038 (95% UI = 41,600–79,449) incident cases, 8,944 (95% UI = 6,150–11,651) deaths, and 529,380 (95% UI = 369,808–675,653) DALYs among the AYAs in China, respectively.

**Figure 1:**
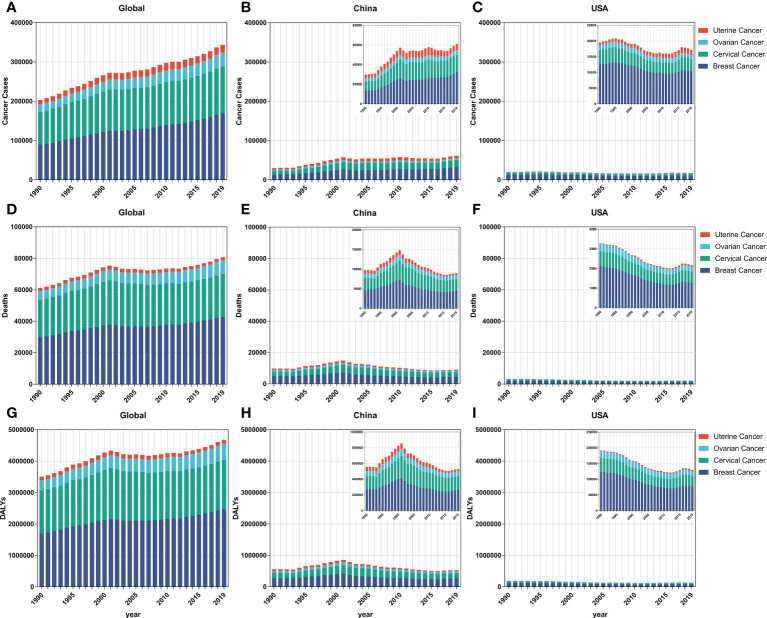
The incidence cases, mortality cases, and DALYs of FeBGCs among AYAs from 1990 to 2019. **(A–C)** The incidence cases of female breast and gynecologic cancers among AYAs in the globe, China, and USA. **(D–F)** The mortality cases of female breast and gynecologic cancers among AYAs in the globe, China, and USA. AYAs, adolescents and young adults; USA, the United States of America. **(G–I)** The DALYs of female breast and gynecologic cancers among AYAs in the globe, China, and USA. AYAs, adolescents, and young adults; DALYs, disability-adjusted life years; FeBGCs, female breast and gynecologic cancers; USA, the United States of America.

As shown in **Figure 1**, incident cases of breast (89.3%), ovarian (86.4%), and uterine (71.3%) cancers increased globally and among the AYAs between 1990 and 2019. In China, the overall incidence (66.4%), mortality cases (41.8%), and DALYs (41.5%) were on a rapid increase from 1990 to 2001, and relatively stable tendency with a slight rise in incidence cases (5.8%) but a decrease in mortality cases (38.1%) and DALYs (36.3%) from 2002 to 2019. Specifically, for breast cancer, although the number of incidence cases increased (10.0%), the number of deaths (38.2%) and DALYs (35.7%) declined from 2002 to 2019 in China. Fortunately, all the overall incident and mortality cases and DALYs in the FeBGCs except for uterine cancer in the USA decreased in the past three decades (**Figures 1C, F, I**). On the whole, the numbers of new incidences, deaths, and DALYs of FeBGCs in China were more than those in the USA each year, and breast and cervical cancers were the top two burdens of FeBGCs globally, in China, and in the USA.

### The FeBGCs’ incidence, mortality, and DALY burden of the AYAs by age band

**Figure 2:**
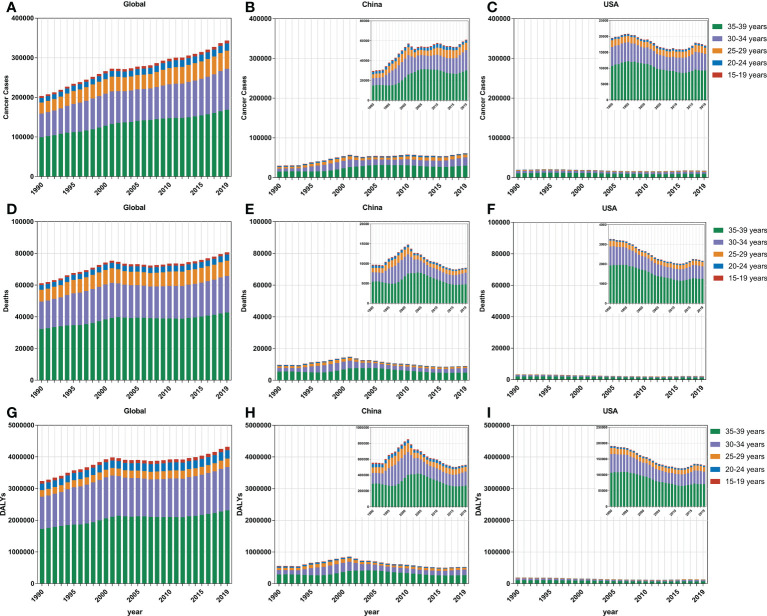
The incidence cases, mortality cases, and DALYs of FeBGCs among AYAs by age. **(A–C)** The incidence cases of female breast and gynecologic cancers among AYAs in the globe, China, and USA. **(D–F)** The mortality cases of female breast and gynecologic cancers among AYAs in the globe, China, and USA. **(G–I)** The DALYs of female breast and gynecologic cancers among AYAs in the globe, China, and USA. AYAs, adolescents and young adults; DALYs, disability-adjusted life years; FeBGCs, female breast and gynecologic cancers; USA, the United States of America.

**Figure 3:**
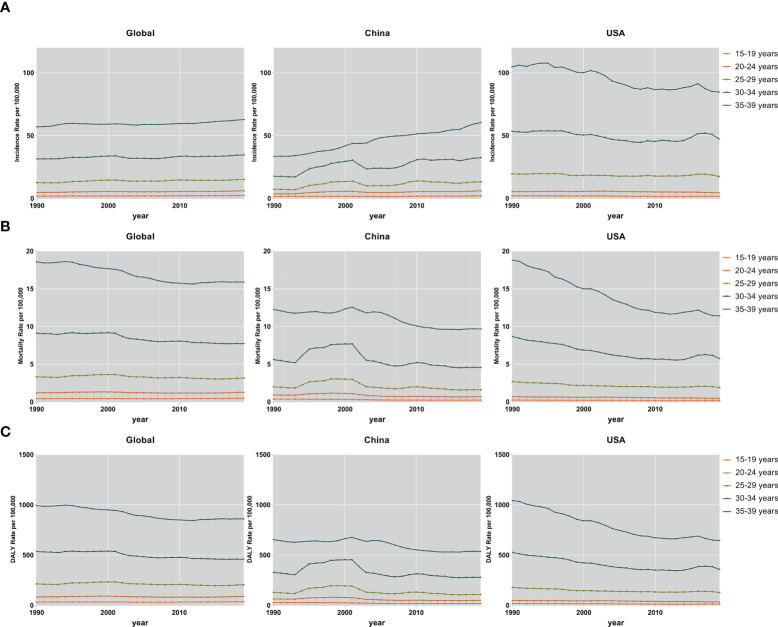
The incidence, mortality, and DALY rates of FeBGCs among AYAs. The incidence **(A)**, mortality **(B)**, and DALY **(C)** rates of female breast and gynecologic cancers among AYAs in the globe, USA, and China, from 1990 to 2019. AYAs, adolescents and young adults; DALY, disability-adjusted life years; FeBGCs, female breast and gynecologic cancers; USA, the United States of America.

Subjects aged 30–34 and 35–39 years accounted for the majority of the FeBGC population in morbidity and mortality for the AYAs worldwide, in China, and in the USA. In 2019, the incidence cases of women aged 30 to 39 years were higher than those of women between 15 and 29 years (globe, 3.8-fold, China, 4.9-fold) globally and in China (**Figure 2**). Interestingly, the increase in incidence and the decline in mortality and DALY of FeBGCs achieved congruence in China, the USA, and the world. In 2019, approximately 20,718 (95% UI = 13,968–27,095) and 29,914 (95% UI = 20,392–38,956) new cases and 2,908 (95% UI =1,982–3,794) and 4,789 (95% UI =3,277–6,220) deaths caused by FeBGCs in China in the age bands of 30–34 and 35–39 years, respectively, and only 739 (95% UI = 522–940) cases and 89 (95% UI = 62–113) mortality occurred in Chinese FeBGCs aged 15–19 years. As shown in **Figure 3**, from 1990 to 2019, the morbidity of FeBGCs held a stable change for each age group, and the mortality and DALY rates decreased globally. In China, the mortality and DALY rates decreased with a few fluctuations after 2001, although the incidence rate increased obviously for the FeBGCs in the 30–34-year (6.5%) and 35–39-year (37.6%) bands. Despite the rates of incidence, mortality, and DALY being higher in the USA, the trends for the FeBGCs in the 30–34-year (incidence, decrease 11.2%, death, decrease 33.8%, DALY, decrease 32.2%) and 35–39-year (incidence, decrease 19.1%, death, decrease 39.3%, DALY, decrease 38.0%) bands decreased more significantly than those in the 30–34-year (incidence, increase 84.4%, death, decrease 18.7%, DALY, decrease 15.0%) and 35–39-year (incidence, increase 80.5%, death, decrease 21.2%, DALY, decrease 17.7%) bands in China between 1990 and 2019.

In 2019, the incidence rates were higher in patients aged 30–39 years than in those aged 15–29 years for breast cancer (5.3-fold), cervical cancer (4.3-fold), ovarian cancer (1.5-fold), and uterine cancer (4.4-fold) globally (**Figure S1**). The age-specific incidence rate of uterine cancer had grown over that of ovarian cancer in the 30–34-year (China, 1.3-fold, USA, 1.5-fold) and 35–39-year (China, 1.6-fold, USA, 1.7-fold) age bands in China and the USA (**Figure S1A**). The age-standardized rates of incidence, death, and DALY in breast and cervical cancers were generally higher for each 5-year age group in the USA than those in China, despite a decreasing trend between 1990 and 2019 (**Figures S2, S3, S4**). In comparison with the AYAs with breast cancer in 1990 in the USA, in 2019, the age-standardized incidence rate (ASIR) decreased to 74.4%, the age-standardized mortality rate (ASMR) decreased to 55.0%, and the age-standardized DALY rate (ASDR) decreased to 56.5% among the 35–39-year bands, while in China, when compared with the AYAs with breast cancer in 1990, in 2019, the ASIR increased to 1.95-fold, but the ASMR decreased to 74%, and the ASDRs decreased to 78.7%. Moreover, the burdens of death and DALY due to uterine cancer were higher in China than those in the USA and the globe in the past 30 years (**Figures S3D, S4D**). In total, in each age group, the burdens of incidences, deaths, and DALYs were the heaviest in breast and cervical cancers.

### The FeBGCs’ age-standardized incidence, mortality, and DALY burden on the AYAs

As shown in **Table 1** and **Figure 1**, for the AYAs with FeBGCs, breast cancer was the most commonly diagnosed cancer and the leading cause of cancer-related death worldwide, followed by cervical, ovarian, and uterine cancers, respectively. The ASIRs of breast cancer in China increased by 5.9% from 1990 to 2019, with the estimated annual percentage change (EAPC) value of 2.28 (95% CI = 2.00–2.57, China), which was higher than -0.97 (95% CI = -1.13 to -0.82) in the USA, -0.19 (95% CI = -0.25–0.12) in the high-SDI region, 1.35 (95% CI =1.29–1.42) in the low-SDI region, and 2.08 (95% CI = 1.09–3.08) in the low-middle-SDI region, 0.60 (95% CI = 0.50–0.69) on a global scale, indicating that the growth rate of breast cancer in China was higher than that in the USA and even the world in the past 30 years. Moreover, the EAPC values of 1.84 (95% CI = 1.49–2.19), 0.66 (95% CI = 0.37–0.95), and 1.33 (95% CI = 0.76–1.91) in cervical, ovarian, and uterine cancers, respectively, demonstrated that the ASIRs of corresponding cancers in China were on an upward trajectory between 1990 and 2019.

**Table 1:** The incidence, mortality, DALYs, and age-standardized rates, and their temporal trends of FeBGCs among AYAs.

Locations	Incidence					Deaths					DALYs				
	1990		2019		1990-2019	1990		2019		1990-2019	1990		2019		1990-2019
	CasesNo. ×10³ (95% UI)	ASIR per 100,000No. (95% UI)	CasesNo. ×10³ (95% UI)	ASIR per 100,000No. (95% UI)	EAPC(95% CI)	CasesNo. ×10³ (95% UI)	ASMR per 100,000No. (95% UI)	CasesNo. ×10³ (95% UI)	ASMR per 100,000No. (95% UI)	EAPC(95% CI)	DALYsNo. ×10³ (95% UI)	ASDR per 100,000No. (95% UI)	DALYsNo. ×10³ (95% UI)	ASDR per 100,000No. (95% UI)	EAPC(95% CI)
Breast cancer															
Global	89.2 (83.7-94.7)	9.0 (8.5-9.6)	168.8 (152.1-186.4)	11.3 (10.2-12.5)	0.60 (0.50-0.69)	29.8 (27.4-32.2)	3.0 (2.8-3.3)	42.7 (38.5-47.3)	2.9 (2.6-3.2)	-0.41 (-0.52 to -0.30)	1692.5 (1561.1-1820.7)	171.1 (157.6-184.9)	2468.5 (2238.0-2701.5)	165.2 (148.5-182.3)	-0.34 (-0.46 to -0.23)
China	13.0 (10.2-16.2)	5.4 (4.2-6.7)	32.3 (24.3-41.4)	11.3 (8.5-14.6)	2.28 (2.00-2.57)	4.7 (3.7-5.9)	2.0 (1.5-2.5)	4.5 (3.4-5.7)	1.6 (1.2-2.0)	-1.44 (-1.80 to -1.08)	266.7 (211.7-328.1)	110.4 (87.2-137.4)	265.9 (207.7-330.6)	93.2 (72.0-116.8)	-1.23 (-1.59 to -0.86)
USA	12.5 (11.9-13.2)	22.3 (21.1-23.5)	10.3 (8.0-13.2)	17.5 (13.7-22.5)	-0.97 (-1.13 to -0.82)	2.1 (2.0-2.2)	3.7 (3.6-3.9)	1.3 (1.2-1.4)	2.2 (2.0-2.3)	-1.99 (-2.21 to -1.77)	123.2 (118.8-127.8)	218.8 (209.3-228.9)	76.5 (70.8-82.7)	130.5 (119.4-142.3)	-1.87 (-2.09 to -1.66)
SDI															
High	29.6 (28.4-31.0)	17.5 (16.8-18.3)	31.4 (27.6-35.4)	17.0 (15.0-19.2)	-0.19 (-0.25 to -0.12)	5.5 (5.4-5.6)	3.3 (3.2-3.3)	3.7 (3.5-4.0)	2.0 (1.9-2.1)	-1.81 (-1.94 to -1.67)	318.5 (308.8-329.0)	188.6 (182.3-195.7)	225.3 (212.4-239.9)	122.1 (114.2-130.7)	-1.65 (-1.78 to -1.52)
High-middle	23.3 (21.8-25.0)	9.9 (9.2-10.6)	39.2 (34.1-45.3)	13.3 (11.6-15.4)	0.92 (0.80-1.03)	6.9 (6.5-7.5)	2.9 (2.7-3.2)	6.8 (6.0-7.5)	2.3 (2.0-2.6)	-1.21 (-1.37 to -1.05)	392.8 (370.2-419.2)	166.4 (155.4-179.2)	393.8 (355.6-433.6)	133.7 (119.9-148.4)	-1.09 (-1.24 to -0.93)
Middle	21.8 (19.2-24.5)	6.8 (6.0-7.7)	55.1 (48.2-62.7)	11.3 (9.9-12.8)	1.55 (1.44-1.66)	9.2 (8.2-10.4)	2.9 (2.6-3.3)	14.0 (12.4-15.9)	2.9 (2.5-3.2)	-0.24 (-0.35 to -0.14)	522.2 (467.5-581.6)	164.2 (146.2-184.8)	804.9 (713.6-901.1)	164.7 (145.2-185.6)	-0.17 (-0.28 to -0.06)
Low-middle	10.7 (8.8-12.7)	5.7 (4.7-6.7)	30.0 (25.2-35.7)	8.7 (7.3-10.3)	2.08 (1.09-3.08)	5.8 (4.8-7.0)	3.1 (2.6-3.7)	11.9 (9.8-14.2)	3.4 (2.9-4.1)	0.11 (-0.02-0.24)	330.1 (278.5-383.8)	175.3 (145.4-210.5)	681.2 (580.8-794.4)	197.2 (163.3-236.3)	0.16 (0.03-0.29)
Low	3.7 (3.0-4.4)	4.5 (3.7-5.3)	13.0 (10.6-15.6)	6.8 (5.5-8.1)	1.35 (1.29-1.42)	2.3 (1.9-2.7)	2.8 (2.3-3.3)	6.3 (5.2-7.6)	3.4 (2.8-4.0)	0.51 (0.44-0.57)	127.6 (107.1-151.0)	156.1 (129.0-187.1)	361.0 (303.7-426.3)	189.0 (156.5-226.1)	0.54 (0.48-0.60)
Cervical cancer															
Global	83.4 (71.9-97.2)	8.3 (7.2-9.7)	119.3 (100.0-135.5)	8.0 (6.7-9.0)	-0.23 (-0.28 to -0.18)	24.0 (20.1-28.3)	2.4 (2.0-2.8)	27.2 (22.9-31.3)	1.8 (1.5-2.1)	-1.09 (-1.16 to -1.02)	1371.7 (1154.6-1594.9)	137.3 (115.6-162.1)	1560.5 (1321.9-1781.7)	104.3 (87.9-120.3)	-1.07 (-1.14 to -0.99)
China	9.4 (6.7-17.7)	3.9 (2.7-7.3)	17.4 (9.4-23.2)	6.1 (3.3-8.2)	1.84 (1.49-2.19)	3.0 (2.1-5.4)	1.2 (0.9-2.2)	2.9 (1.7-3.9)	1.0 (0.6-1.4)	-0.58 (-0.96 to -0.21)	172.0 (125.1-300.4)	70.3 (50.3-125.9)	169.2 (96.3-221.0)	59.8 (33.9-78.5)	-0.54 (-0.92 to -0.16)
USA	4.3 (3.4-4.7)	7.8 (6.1-8.5)	3.8 (2.8-4.9)	6.6 (4.9-8.5)	-0.78 (-0.98 to -0.57)	0.8 (0.6-0.8)	1.4 (1.0-1.5)	0.6 (0.5-0.6)	1.0 (0.8-1.1)	-1.24 (-1.48 to -1.01)	44.1 (34.3-46.7)	79.0 (61.1-85.0)	33.0 (28.6-36.4)	56.8 (48.7-63.9)	-1.21 (-1.45 to -0.98)
SDI															
High	14.1 (11.6-15.0)	8.4 (6.9-8.9)	11.6 (9.8-13.4)	6.4 (5.4-7.4)	-0.89 (-1.01 to -0.77)	2.2 (1.8-2.4)	1.3 (1.1-1.4)	1.4 (1.2-1.5)	0.8 (0.7-0.8)	-1.93 (-2.12 to -1.74)	130.0 (107.0-136.3)	77.3 (63.5-81.9)	82.7 (73.6-89.1)	45.5 (40.0-49.7)	-1.85 (-2.03 to -1.66)
High-middle	16.5 (15.1-19.7)	7.0 (6.4-8.3)	21.9 (17.0-25.5)	7.5 (5.8-8.7)	0.31 (0.25-0.37)	4.1 (3.7-5.0)	1.7 (1.6-2.1)	3.8 (3.0-4.3)	1.3 (1.0-1.5)	-1.11 (-1.21 to -1.02)	233.2 (214.1-282.8)	98.6 (89.9-119.7)	217.8 (172.8-244.8)	74.5 (59.5-84.8)	-1.06 (-1.15 to -0.96)
Middle	23.9 (20.4-30.0)	7.4 (6.3-9.3)	34.1 (27.2-39.6)	7.0 (5.6-8.1)	-0.36 (-0.50 to -0.23)	7.2 (6.1-9.0)	2.3 (1.9-2.8)	7.1 (5.8-8.2)	1.4 (1.2-1.7)	-1.71 (-1.86 to -1.55)	412.7 (355.2-512.8)	127.9 (108.8-160.1)	406.1 (330.8-462.2)	83.1 (68.2-96.0)	-1.67 (-1.83 to -1.51)
Low-middle	17.6 (13.5-21.8)	9.3 (7.2-11.5)	30.5 (24.8-36.9)	8.8 (7.2-10.7)	-0.30 (-0.39 to -0.22)	6.1 (4.6-7.6)	3.3 (2.5-4.1)	8.1 (6.5-10.0)	2.4 (1.9-2.9)	-1.22 (-1.31 to -1.12)	348.4 (272.0-419.3)	184.5 (141.0-230.6)	465.0 (381.0-552.2)	135.1 (108.4-165.7)	-1.19 (-1.29 to -1.10)
Low	11.2 (8.0-14.2)	13.6 (9.8-17.3)	21.0 (15.8-27.2)	11.0 (8.3-14.3)	-0.84 (-0.88 to -0.79)	4.3 (3.1-5.5)	5.3 (3.8-6.8)	6.8 (5.2-8.8)	3.6 (2.8-4.7)	-1.49 (-1.54 to -1.43)	246.2 (174.8-308.4)	300.2 (212.0-383.5)	387.5 (296.3-498.2)	204.2 (155.2-264.8)	-1.47 (-1.53 to -1.42)
Ovarian cancer															
Global	19.2 (15.9-24.3)	1.9 (1.5-2.3)	35.8 (30.1-41.4)	2.4 (2.0-2.8)	0.74 (0.66-0.83)	5.4 (4.4-7.2)	0.5 (0.4-0.7)	8.9 (7.4-10.3)	0.6 (0.5-0.7)	0.19 (0.09-0.30)	323.6 (260.4-428.7)	31.4 (25.3-41.4)	529.0 (442.6-602.4)	35.7 (29.6-41.2)	0.22 (0.12-0.33)
China	3.5 (2.3-4.8)	1.4 (0.9-1.9)	5.1 (3.7-7.0)	2.0 (1.4-2.7)	0.66 (0.37-0.95)	1.1 (0.7-1.5)	0.4 (0.3-0.6)	1.1 (0.8-1.5)	0.4 (0.3-0.6)	-0.93 (-1.26 to -0.61)	64.7 (44.6-89.5)	25.4 (17.2-35.3)	65.6 (46.7-87.0)	24.8 (17.7-33.0)	-0.90 (-1.23 to -0.56)
USA	1.8 (1.6-1.9)	3.3 (3.1-3.6)	1.5 (1.1-1.9)	2.6 (1.9-3.4)	-0.88 (-0.99 to -0.77)	0.4 (0.3-0.4)	0.7 (0.6-0.7)	0.3 (0.2-0.3)	0.5 (0.4-0.5)	-1.07 (-1.46 to -0.68)	21.1 (20.2-22.1)	38.9 (36.0-41.6)	15.7 (14.3-17.8)	27.4 (24.4-32.2)	-1.18 (-1.25 to -1.11)
SDI															
High	5.2 (4.8-5.5)	3.2 (2.9-3.3)	5.0 (4.3-5.7)	2.9 (2.5-3.3)	-0.38 (-0.46 to -0.30)	1.1 (1.0-1.2)	0.7 (0.6-0.7)	0.9 (0.8-1.0)	0.5 (0.4-0.5)	-1.16 (-1.21 to -1.11)	66.1 (61.7-68.5)	40.0 (36.5-42.1)	51.9 (47.7-56.5)	29.6 (26.9-33.1)	-1.11 (-1.16 to -1.06)
High-middle	6.0 (4.9-6.7)	2.5 (2.1-2.8)	7.9 (6.5-9.1)	2.9 (2.4-3.4)	0.29 (0.19-0.40)	1.6 (1.3-1.9)	0.7 (0.6-0.8)	1.7 (1.4-2.0)	0.6 (0.5-0.7)	-0.70 (-0.84 to -0.56)	96.3 (78.9-108.8)	40.6 (33.2-46.2)	102.1 (85.8-114.5)	37.1 (30.8-42.3)	-0.66 (-0.80 to -0.52)
Middle	5.4 (4.0-7.2)	1.5 (1.1-2.1)	12.1 (9.6-14.5)	2.6 (2.0-3.1)	1.38 (0.81-1.95)	1.7 (1.2-2.2)	0.5 (0.4-0.7)	2.9 (2.3-3.4)	0.6 (0.5-0.7)	0.46 (0.34-0.59)	100.4 (74.2-134.9)	29.2 (21.5-39.2)	174.3 (139.1-202.5)	36.6 (28.9-43.1)	0.49 (0.37-0.62)
Low-middle	2.0 (1.4-3.6)	1.0 (0.7-1.8)	7.6 (5.8-9.9)	2.1 (1.6-2.8)	2.69 (2.64-2.75)	0.7 (0.5-1.3)	0.4 (0.3-0.7)	2.3 (1.7-3.0)	0.6 (0.5-0.8)	1.97 (1.92-2.01)	42.9 (30.5-78.4)	21.3 (15.0-38.8)	134.7 (105.0-170.3)	38.1 (28.6-50.3)	1.98 (1.94-2.03)
Low	0.7 (0.5-1.7)	0.8 (0.5-1.9)	3.3 (2.5-4.2)	1.6 (1.2-2.0)	2.27 (2.19-2.35)	0.3 (0.2-0.7)	0.3 (0.2-0.8)	1.1 (0.9-1.4)	0.4 (0.2-0.8)	1.59 (1.50-1.67)	17.7 (11.4-41.3)	20.2 (12.6-47.3)	65.6 (52.3-81.9)	32.4 (25.1-41.7)	1.60 (1.52-1.69)
Uterine cancer															
Global	11.3 (8.9-12.8)	1.4 (1.1-1.6)	19.4 (15.5-22.2)	1.7 (1.3-1.9)	0.41 (0.22-0.60)	1.9 (1.4-2.3)	0.2 (0.2-0.3)	1.8 (1.4-2.0)	0.2 (0.1-0.2)	-1.99 (-2.24 to -1.73)	114.1 (79.1-134.0)	14.5 (10.1-17.3)	110.2 (85.1-124.2)	9.4 (7.1-10.7)	-1.83 (-2.08 to -1.57)
China	3.6 (2.0-4.9)	1.9 (1.1-2.5)	6.2 (3.9-8.3)	2.8 (1.8-3.8)	1.33 (0.76-1.91)	0.9 (0.5-1.2)	0.5 (0.3-0.6)	0.5 (0.3-0.6)	0.2 (0.1-0.3)	-3.20 (-3.77 to -2.64)	52.0 (28.9-67.4)	26.9 (15.0-35.7)	28.7 (19.1-37)	13.1 (8.6-17.1)	-2.91 (-3.47 to -2.35)
USA	0.8 (0.8-0.9)	1.9 (1.8-2.1)	1.6 (1.2-2.1)	3.5 (2.7-4.5)	2.42 (2.29-2.55)	<0.1 (<0.1-<0.1)	0.1 (0.1-0.1)	0.1 (0.1-0.1)	0.1 (0.1-0.2)	1.64 (1.50-1.77)	2.8 (2.6-3.1)	6.4 (5.8-7.0)	4.4 (3.9-4.9)	9.5 (8.4-10.8)	1.79 (1.65-1.92)
SDI															
High	2.4 (2.2-2.6)	1.8 (1.7-2.0)	4.0 (3.5-4.6)	2.8 (2.4-3.2)	1.88 (1.74-2.03)	0.2 (0.1-0.2)	0.1 (0.1-0.1)	0.2 (0.2-0.2)	0.1 (0.1-0.1)	0.13 (-0.04-0.30)	10.6 (9.2-11.5)	8.0 (6.9-8.9)	11.4 (10.4-12.4)	7.9 (7.1-8.8)	0.40 (0.24-0.56)
High-middle	4.6 (4.0-5.2)	2.4 (2.1-2.8)	6.8 (5.5-8.1)	3.0 (2.4-3.6)	0.60 (0.41-0.79)	0.6 (0.5-0.7)	0.3 (0.3-0.4)	0.4 (0.3-0.5)	0.2 (0.1-0.2)	-2.45 (-2.73 to -2.17)	35.2 (28.2-40.4)	18.9 (15.1-22.2)	26.2 (21.4-30.1)	11.5 (9.3-13.5)	-2.15 (-2.42 to -1.88)
Middle	3.3 (1.9-4.1)	1.3 (0.7-1.6)	6.0 (3.9-7.3)	1.6 (1.0-1.9)	0.79 (-0.07-1.65)	0.8 (0.5-1.1)	0.3 (0.2-0.4)	0.7 (0.4-0.8)	0.2 (0.1-0.2)	-2.54 (-2.88 to -2.21)	47.4 (26.9-58.9)	18.5 (10.6-23.8)	39.3 (26.2-46.2)	10.3 (6.6-12.2)	-2.39 (-2.73 to -2.06)
Low-middle	0.9 (0.6-1.1)	0.6 (0.4-0.8)	20 (1.5-2.5)	0.7 (0.6-0.9)	0.48 (0.26-0.70)	0.3 (0.2-0.4)	0.2 (0.1-0.2)	0.4 (0.3-0.5)	0.1 (0.1-0.2)	-1.31 (-1.62 to -0.99)	15.6 (9.9-19.9)	10.5 (6.7-13.7)	22.2 (16.7-26.1)	8.2 (6.1-10.2)	-1.25 (-1.57 to -0.94)
Low	0.2 (0.2-0.3)	0.4 (0.2-0.5)	0.7 (0.5-0.9)	0.4 (0.3-0.6)	0.74 (0.69-0.80)	0.1 (0.1-0.1)	0.1 (0.1-0.2)	0.2 (0.1-0.2)	0.1 (0.1-0.2)	-0.49 (-0.55 to -0.43)	5.3 (3.6-7.0)	8.2 (5.6-11.1)	10.9 (8.3-13.7)	7.3 (5.5-9.5)	-0.46 (-0.52 to -0.40)

ASDRs, age-standardized DALY rate; ASIRs, age-standardized incidence rates; ASMRs, age-standardized mortality rates; AYAs, adolescents and young adults; DALY, disability-adjusted life year; EAPCs, estimated annual percentage change; FeBGCs, female breast and gynecologic cancers; SDI, social-demographic index; USA, the United States of America.

As shown in **Table 1**, nearly all the EAPC values of negative numbers (<0) indicated that the ASMRs and ASDRs in breast cancer decreased around the world except for the low-SDI regions (EAPC value in ASMR, 0.51, 95% CI = 0.44–0.57, EAPC value in ASDR, 0.54, 95% CI = 0.48–0.60) and low-middle SDI regions with the EAPC values of 0.11 (EAPC value in ASMR, 0.11, 95% CI = -0.02–0.24, EAPC value in ASDR, 0.16, 95% CI = 0.03–0.29). In China, the EAPC values of -1.44 (95% CI = -1.80 to -1.08) and -1.23 (95% CI = -1.59 to -0.86) revealed that the declines in ASMR and ASDR were more rapid than those in the world (EAPC value in ASMR, -0.41, 95% CI = -0.52 to -0.30, EAPC value in ASDR, -0.34, 95% CI =-0.46 to -0.23) and slower than those in USA (EAPC value in ASMR, -1.99, 95% CI = -2.21 to -1.77, EAPC value in ASDR, -1.87, 95% CI = -2.09 to -1.66). Moreover, the ASDR per 100,000 of 135.1 (95% UI = 108.4–165.7) in the low-SDI region and 204.2 (95% UI = 155.2–264.8) in the low-middle-SDI region demonstrated that the burden attributed to cervical cancer in China was heavier than that in other countries and regions in 2019. In China, the ASMR and ASDR per 100,000 values of uterine cancer were 0.3 in 1990 and 0.2 in 2019, and 18.9 in 1990 and 11.5 in 2019, respectively, which demonstrated that the ASMR and ASDR per 100,000 of uterine cancer in China were higher than those in the globe (ASMR, from 0.1 in 1990 to 0.1 in 2019, ASDR, from 8.0 in 1990 to 7.9 in 2019) in the past 30 years.

### Risk factors attributable to the FeBGCs’ burden

**Figure 4:**
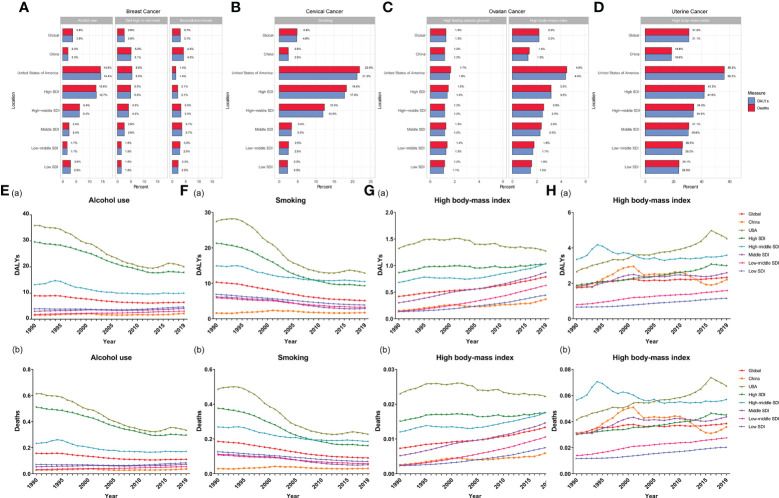
Percentage of factors for FeBGCs among AYAs in 2019 and attributive rates in 1990–2019. **(A)** Percentage of alcohol use, diet high in red meat, and secondhand smoke for female breast cancer; **(B)** percentage of smoking and unsafe sex for cervical cancer; **(C)** percentage of high fasting plasma and high body mass index for ovarian cancer; **(D)** percentage of high body mass index for uterine cancer; **(E)** rates of DALYs **(a)** and deaths (b) attributable to alcohol use for breast cancer; **(F)** rates of DALYs (a) and deaths (b) attributable to smoking for cervical cancer; **(G)** rates of DALYs (a) and deaths (b) attributable to high body mass index for ovarian cancer; **(H)** rates of DALYs (a) and deaths (b) attributable to the high body mass index for uterine cancer; ARP, attributable risk percent; AYAs, adolescents and young adults; DALY, disability-adjusted life years; FeBGCs, female breast and gynecologic cancers; SDI, social-demographic index.

The contributions of risk factors to FeBGC deaths and DALYs are demonstrated in **Figure 4**. Expectedly, alcohol use (deaths, 3.8%, DALYs, 3.8%) was the most common contributing factor to mortality and DALYs in breast cancer globally, while the high body mass index (high BMI) contributed mostly to mortality and DALYs to ovarian (deaths, 2.2%, DALYs, 2.2%) and uterine (deaths, 31.2%, DALYs, 31.2%) cancers. Unexpectedly, in China, the major attributive factors of breast cancer mortality and DALYs for the AYAs were diet high in red meat (deaths, 5.2%, DALYs, 5.1%) and secondhand smoke (deaths, 4.3%, DALYs, 4.3%), respectively, followed by alcohol use (deaths, 2.0%, DALYs, 2.0%), while alcohol use contributed 14.3% of mortality and 14.4% of DALYs to the FeBGCs’ burden in the USA (**Figure 4A**). Cervical cancer deaths were mainly attributed to smoking in the USA (deaths, 22.0%; DALYs, 21.3%, **Figure 4B**) than in China (deaths, 2.5%; DALYs, 2.5%, **Figure 4B**). In China, high BMI was correlated with mortality and DALYs for the AYAs with ovarian cancer (deaths, 4.5%; DALYs, 4.4%, **Figure 4C**) and uterine cancer (deaths, 18.8%; DALYs, 18.6%, **Figure 4D**).

The trends of rates of DALYs and deaths attributable to risk factors of FeBGCs are shown in **Figures 4E–H** and **Figure S5**. The rates of DALYs and deaths attributable to alcohol use, the leading attributable factor of breast cancer, had decreased by 43.8% and 45.4%, respectively, in the USA and 39.5% and 42.0%, respectively, in other high-SDI regions, but slightly increased by 41.0% and 31.7%, respectively, in China from 1990 to 2019 (**Figure 4E**). DALYs and deaths of cervical cancer attributable to smoking (DALYs, from 1.62% in 1990 to 1.73% in 2019, deaths, 0.30‰ to 0.31‰ in 2019, **Figure 4F**) and those of ovarian cancer attributable to high BMI (DALYs, from 0.15% in 1990 to 0.37% in 2019; deaths, 0.03‰ to 0.06‰ in 2019, **Figure 4G**) also increased slightly in China from 1990 to 2019. Moreover, the rates of DALYs and deaths of uterine cancer attributable to high BMI in China (DALYs, from 1.62% in 1990 to 1.73% in 2019; deaths, 0.30‰ to 0.31‰ in 2019, **Figure 4H**) were higher than those in the globe (DALYs, from 1.62% in 1990 to 1.73% in 2019; deaths, 0.30‰ to 0.31‰ in 2019, **Figure 4H**) and showed fluctuations in the past three decades.

### The associations between the EAPC and ASIR

**Figure 5:**
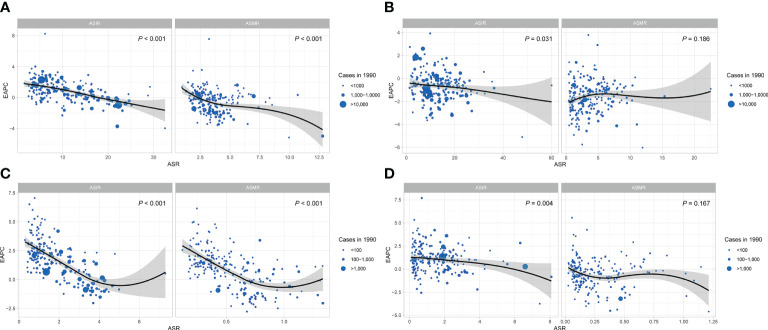
The correlation of EAPC and ASR of FeBGCs among AYAs in 1990. The correlation of EAPC and ASR of female breast cancer **(A)**, cervical cancer **(B)**, ovarian cancer **(C)**, and uterine cancer **(D)** among AYAs in 1990. The size of the blue circle is increased with cancer cases, and one circle represents a specific country or region. The P values were derived from Pearson correlation analysis. The black line represents the average expected relationship between SDIs and ASIRs for female breast and gynecologic cancers based on values from all countries and regions from 1990 to 2019. ASR, age-standardized rate; ASIR, age-standardized incidence rate; ASMR, age-standardized mortality rate; EAPC, estimated annual percentage change; FeBGCs, female breast and gynecologic cancers; SDI, social-demographic index.

The significant negative association detected between the EAPC and ASIR in 1990 for breast cancer (P< 0.001, **Figure 5A**), cervical cancer (P = 0.031, **Figure 5B**) and uterine cancer (P = 0.004, **Figure 5D**) demonstrated that the incidence of breast, cervical and uterine cancers increased more slowly in countries and regions with higher baseline ASIR in 1990. A significant negative correlation was also observed between the EAPC and ASMR (P< 0.001) of breast cancer in 1990, while the EAPC of ovarian cancer and ASIR were negatively correlated when ASIR< 5, and the correlation reversed when ASIR >5 (**Figure 5C**).

### ARIMA model analysis for predicting the morbidities and mortalities of FeBGCs among AYAs in China

**Figure 6:**
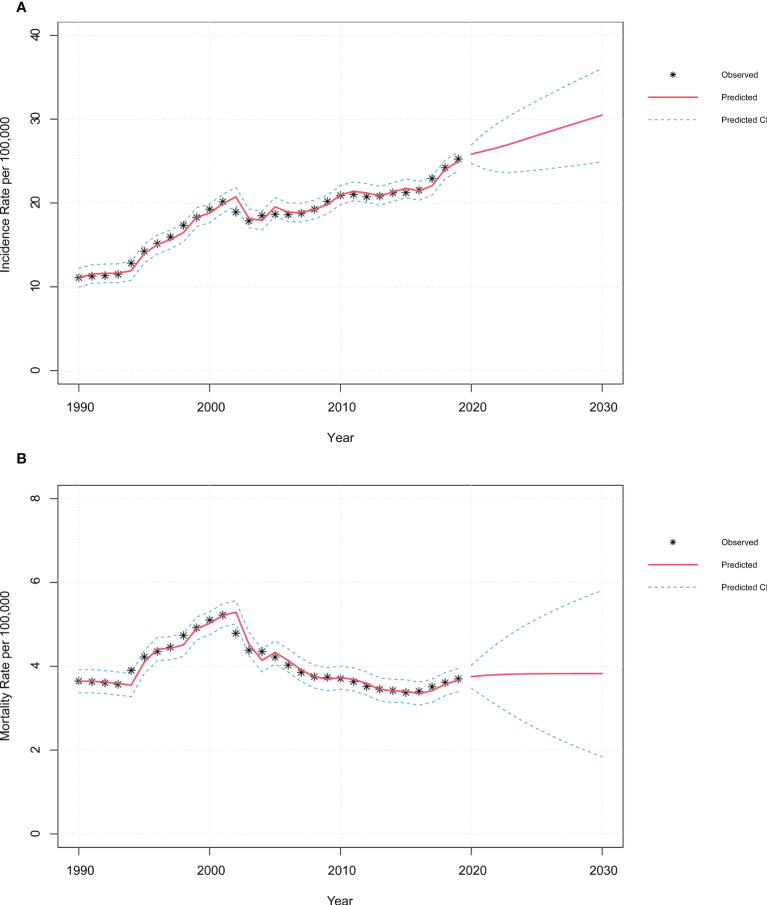
|Time-series plots of incidence and mortality of FeBGCs among AYAs in 1990–2030 in China. Observed and predictive values of incidence **(A)** and mortality **(B)** rates among AYAs from 1990 to 2030 in China. CI, confidence interval; FeBGCs, female breast and gynecologic cancers.

**Figures 6** and **Figure S7** revealed the fitting results of the autoregressive integrated moving average (ARIMA) models of FeBGCs’ incidences among the AYAs in China. Overall, the incidence rate of FeBGCs of the AYAs had increased in the past three decades (2.3-fold) and would be on a continuous increase in China until 2030. In 2030, the incidence rate of FeBGCs would grow to 30.49 per 100,000 (95% CI = 24.93–36.05) in China (**Table 2**), while the mortality rate would maintain a steady state in the years ahead. Breast cancer morbidity will be on the increase (1.3-fold in 2030) for the Chinese AYAs, but the increase would be numerically slower than in the past 10 years (27.9% vs. 38.7% increase, **Figure S6A**). As shown in **Table S1**, the incidence of breast cancer among female AYAs in China would be as high as 17.13 (95% CI = 13.28–20.99) per 100,000 by 2030. Moreover, there would be a slight rise in the morbidity of cervical cancer (1.2-fold in 2030) (**Figure S6B**), while the incidence would maintain a steady progress for ovarian (1.05-fold in 2030) (**Figure S6C**) and uterine cancers 1.07-fold in 2030) (**Figure S6D**) until 2030. As shown in **Figures S6E–H** and **Table S2**, the mortality trend of the FeBGCs would remain stable in the next decade among Chinese AYAs.

**Table 2:** Prediction of incidence and mortality of FeBGCs among AYAs in China in 2020–2030.

Year	Predicted incidence rateper 100,000 (95% CI)	Predicted mortality rateper 100,000 (95% CI)
2020	25.83 (24.71-26.95)	3.75 (3.48-4.03)
2021	26.21 (24.06-28.36)	3.78 (3.26-4.30)
2022	26.60 (23.70-29.50)	3.80 (3.06-4.54)
2023	27.05 (23.64-30.46)	3.81 (2.86-4.76)
2024	27.55 (23.75-31.35)	3.82 (2.68-4.95)
2025	28.05 (23.92-32.18)	3.82 (2.51-5.13)
2026	28.55 (24.10-32.99)	3.82 (2.36-5.29)
2027	29.03 (24.29-33.78)	3.82 (2.22-5.43)
2028	29.52 (24.48-34.55)	3.83 (2.09-5.57)
2029	30.00 (24.70-35.31)	3.83 (1.96-5.69)
2030	30.49 (24.93-36.05)	3.83 (1.84-5.81)

CIs, confidence intervals; FeBGCs, female breast and gynecologic cancers.

The fitting processes of the models are exhibited in **Figures S7, S8, and S9**, and the goodness-of-fit tests are shown in **Tables S2–S4**, with three alternative models shown for each fit. Time-series plots indicated that the original time sequences were unstable and became stable after differential transformations (**Figures S7–S9**). In the models we built, the log-likelihood values were relatively large, while the Akaike information criterion (AIC) values were relatively small, and there were statistically significant differences in the parameter. Therefore, the models built in the current study were optimal and credible within our observation time.

## Discussion

To promote women’s health and reduce the female breast and gynecologic cancer (FeBGC) burden of the AYAs in China, this GBD-based study, which discloses the most up-to-date trends and patterns of the incidence, mortality, and disability-adjusted life years (DALYs) associated with FeBGCs worldwide and the most relevant risk factors, was performed. In this study, our analysis demonstrated that with the 343,281 global new cases of FeBGCs in 2019, the Chinese incident cases of FeBGCs rapidly increased from 1990 to 2001 but slightly rose between 2002 and 2019. Such results are identical to those reported in the literature based on the National Central Cancer Registry of China (21, 22). Moreover, this is the first study on Chinese AYAs with female cancers to predict morbidity and mortality using time-series analysis.

According to previous studies based on the global cancer burden, the largest increase in cancer incident cases occurred in middle-SDI countries (52% increase), and the changes in FeBGC incident cases were mainly attributable to population growth (10%), changing age structure (24%%), and age-specific incidence rates (18%) (1). In China, the natural population growth rate, which is as high as 14.39% in 1990, tended to decrease dramatically to 6.45% since 2002 with the implementation of the one-child policy (16). Moreover, the Westernization of lifestyle particularly rises in the prevalence of obesity and loss of physical activity in recent decades in China is likely to have had a significant effect on the incidence of the FeBGCs for the AYAs in China in the past three decades with a high speed. In addition, the change in sexual attitudes and behaviors in China is also a factor that cannot be neglected in the occurrence of cervical cancer. For these reasons, the FeBGC incident cases have a continuously increasing tendency and will be increased by 1.3-fold in 2030, with the estimated annual percentage change (EAPC) value being greater than zero.

Research based on GBD data confirms that, in 2020, the number of female breast cancers has surpassed that of lung cancer as the most commonly diagnosed cancer in the world, with an estimated 2.3 million new cases (23). In China, the median age of breast cancer at diagnosis was younger than that in the USA (China vs. the USA, 48–50 vs. 64 years) (24). In our research, the crude incidence rate of breast cancer in Chinese AYAs had increased from 4.88 per 100,000 in 1990 to 13.36 per 100,000 in 2019, and it would continue to rise to 17.13 per 100,000 by 2030, with women aged 30–39 accounting for the largest cancer burden. More importantly, in China, the public policy of free two-cancer screening, the screening of female breast and cervical cancers, in rural areas has been launched for more than 10 years and had an impact on the observed rise in the FeBGCs for the AYAs.

Mortality and disability-adjusted life years (DALYs) are important measurements to assess the global burden of this disease. In our study, the mortality and DALYs of the FeBGCs for the AYAs decreased between 1990 and 2021. Moreover, the trends achieved congruence in the USA and the world. Such results, which are similar to the previous research that the cancer death rate has fallen continuously from 1991 through 2018 (25, 26), are inconsistent with the research in China with an increasing trend in mortality in breast, cervix, and ovary cancers (21, 22). The underlying reason could contribute to reductions in the prevalence of risk factors and improvements in early detection and treatment, particularly for migrant workers in cities and those living in rural areas and disadvantaged populations. With the early-staged FeBGCs and standardized comprehensive treatment, the mortality and DALYs of the FeBGCs for the Chinese AYAs had been significantly decreasing in the past 30 years (EAPC = -1.44, 95% CI -1.80 to -1.08). Moreover, these results indirectly indicate that, in China, we should pay more attention to the screening projects, with the aim to reduce the burden of the FeBGCs for the AYAs.

As the fourth most common cancer worldwide and the most common cancers in women aged 15–44, the tumorigenesis of cervical cancer always experiences more than a decade of persistent infection with the high-risk human papillomavirus (HPV). Moreover, cervical cancer is often diagnosed at a young age (27, 28), and the morbidity of cervical cancer is the highest among gynecologic cancers in China (27). The prevention and control of cervical cancer play the largest role in easing the disease burden of FeBGCs through HPV vaccination and cancer screening in young women. Increasing evidence indicates that, according to a predictive model built in 2019 (29), the vaccination starting at the age of 12 would contribute to the elimination of cervical cancer in the 2070s in China. However, the promotion of these prevention approaches requires adequate public health resources supported by one country or region. In the current study, the burden of cervical cancer is much heavier in the low- and low-middle SDI regions in comparison with that in the high-SDI region in 2019 (low SDI, ASDR = 204.2 per 100,000, low-middle SDI, ASDR = 135.1 per 100,000, high SDI, ASDR = 45.5 per 100,000). As a representative of the developing countries, in China, an increasing tendency in the morbidity of cervical cancer is found in the female AYAs in the past three decades (EAPC = 1.84, 95% CI = 1.49 to 2.19). Fortunately, the HPV vaccination and two cancer screenings have been supported by the Chinese government among young women and implemented in the rural areas and even on a national scale.

Ovarian and uterine cancers are not common cancers among young women (30, 31). However, impaired fertility after standard staging and treatment of the two cancers is a major concern for young female patients. In our research, the incidence of ovarian and uterine cancers had increased in most countries and regions except for the high-SDI region represented by the USA. Moreover, the incidence would maintain gentle progress for ovarian (1.05-fold) and uterine cancers (1.07-fold) in 2030 through ARIMA model analysis. Obesity and estrogen without progesterone antagonism are considered to be importantly common risk factors for ovarian, uterine, and endometrial cancers (9, 32). Obesity caused by modern diet and lifestyle, changes in hormone levels affected by obesity, and delayed childbirth among young women would contribute to the increase in morbidity of ovarian and uterine cancers (31). In addition, we know that ovarian cancer is a leading cause of death among gynecologic cancers, due to the lack of appropriate prevention and screening methods (33). Fortunately, in the current study, through ARIMA model analysis, the mortality rates of ovarian and uterine cancers would not increase in 2030. Although screening of ovarian and uterine cancers is not recommended for women without clinical symptom (33), weight loss and lifestyle changes are feasible prevention methods for young women.

Unhealthy lifestyles in China, such as excessive tobacco use and alcohol consumption, obesity, and incorrect components of the diet, have contributed to the increasing risk of cancer (34). Expectedly, in China, diet high in red meat was the most common contributing factor to mortality and DALYs in breast cancer, followed by secondhand smoke, followed by alcohol use, while alcohol use contributed mostly to the mortality and DALYs to the FeBGCs’ burden in the USA. Such results were concordant with the previous research that secondhand smoke and red meat consumption were associated with an increased risk of breast cancer mortality and incidence (35–37). The current study also found that cervical cancer mortalities and DALYs could be mainly attributed to smoking among young Chinese women. Moreover, the findings in the current study further confirmed the impact of Westernization on diet structure and tobacco use in family and social places that contributed mostly to the FeBGCs’ burden in China. Therefore, it is imperative to enterprise tobacco control policies and change in dietary patterns from diet high in red meat to fruits and vegetables. Moreover, the policy-making of smoking cessation that reduces secondhand smoke exposure and a green modern diet that reduces the intake of red meat could have an important impact on reducing the breast and cervical cancer burden in China (35, 36).

Moreover, early research confirmed that increasing BMI was also identified as a potential risk factor attributable to the increase in the incidence and mortality of ovarian and uterine cancers (38–40). Consistently, in the current study, high BMI has contributed mostly to mortality and DALYs to ovarian and uterine cancers. As previous studies reported, obesity is associated with high levels of insulin, and women with endometrial cancer have an increased endometrial expression of genes involved in the insulin signaling pathway (41, 42). Therefore, the insulin and insulin-like growth factor axis and adipokines are always regarded to be the most studied candidates, although mechanisms that link high BMI and risk of ovarian and uterine cancers are not fully understood (39). In addition, the findings in our study showed that up to 19% of deaths and DALYs resulting from uterine cancer could be reduced among the AYAs in China if high BMI could be controlled effectively. Moreover the policy-making of weight control through changing the diet structure and lifestyle that reduces the excess energy could have an important impact on reducing the ovarian and uterine cancer burden in China.

The main strength of the current study is the comprehensive presentation of the FeBGC burden through analyzing the most recent national estimated data worldwide. Nevertheless, this research had some limitations. Firstly, the accuracy of this research was dependent on the quality of GDB data; however, for some regions with poor quality data and incomplete registration, especially some low-SDI regions, the availability of GBD study estimation is poor. Moreover, limited by a low incidence and mortality rate, data on FeBGCs among AYAs are insufficient, especially for women aged 15–29 years, which may affect the accuracy of prediction results. Secondly, the GDB 2019 study did not provide data on uterine cancer in ages 15–19; hence, this research might underestimate the cancer burden of AYAs. Thirdly, we were unable to consider some attributive risk factors when analyzing specific cancer or age groups, which were not provided in the GDB 2019 study. Finally, some critical factors affecting the predictions, such as the COVID-19 pandemic, policy changes in HPV vaccination, and measures for cancer screening, were not taken into account in our predictions, which potentially prevented this prediction from fully meeting future needs, calling for further studies to take more factors into account.

## Conclusion

In conclusion, the incidence of FeBGCs among the AYAs in China had been increasing in the past three decades. Breast and cervical cancer remained a major cancer burden in Chinese female AYAs, and the morbidity would continue to be on the rise until 2030. A diet high in red meat, smoking, and increasing BMI were important contributing factors to mortality and DALYs in breast, cervical and ovarian, and uterine cancers, respectively. In a word, the increasing Chinese FeBGC burden is mainly observed in the AYAs, and non-red meat diet and the control of body weight could reduce FeBGC burden in China.

## Data availability statement

The original contributions presented in the study are included in the article/**Supplementary Material**. Further inquiries can be directed to the corresponding authors.

## Author contributions

YZ and PQ drafted the manuscript and analyzed the data. LY and SL generated the figures. ZY and HZ performed the background research. CZ and JH edited the manuscript. All authors have read and approved the content of the manuscript.

## Funding

This study was supported by the National Natural Science Foundation of China (NSFC 81502413 to CZ) and Shaanxi Provincial Natural Science Foundation of China (SNSFC 2019SF-145 to CZ).

## Acknowledgments

We are thankful to the Institute for Health Metrics and Evaluation for the development of the GBD database. Thanks are due to Boqing Dong for assistance with the R code writing. We thank all our colleagues in the Departments of Breast Surgery, First Affiliated Hospital of Xi’an Jiaotong University.

## Conflict of interest

The authors declare that the research was conducted in the absence of any commercial or financial relationships that could be construed as a potential conflict of interest.

## Publisher’s note

All claims expressed in this article are solely those of the authors and do not necessarily represent those of their affiliated organizations, or those of the publisher, the editors and the reviewers. Any product that may be evaluated in this article, or claim that may be made by its manufacturer, is not guaranteed or endorsed by the publisher.

## Supplementary material

Figure S1 Age-specific numbers and rates of incidence, death, and DALY of FeBGCs among AYAs. Age-specific numbers and rates of Incidence (A), Death (B), and DALY (C) of female breast and gynecologic cancers among AYAs in the globe (a), China(b), and USA (c) in 2019. AYAs, adolescents and young adults; DALY, disability-adjusted life year; FeBGCs, female breast, and gynecologic cancers; USA, the United States of America.

Figure S2 The ASIR of FeBGCs among AYAs. The ASIR of female breast cancer (A), cervical cancer (B), ovarian cancer (C), and uterine cancer (D) among AYAs in the globe, USA, and China, from 1990 to 2019. ASIR, age-standardized incidence rates; AYAs, adolescents and young adults; FeBGCs, female breast and gynecologic cancers; USA, the United States of America. *Data on incidence of uterine cancer of 15-19 years were not available.

Figure S3 The ASMR of FeBGCs among AYAs. The ASMR of female breast cancer (A), cervical cancer (B), ovarian cancer (C), and uterine cancer (D) among AYAs in the globe, USA, and China, from 1990 to 2019. ASMR, age-standardized mortality rates; AYAs, adolescents and young adults; FeBGCs, female breast and gynecologic cancers; USA, the United States of America. *Data on mortality of uterine cancer of 15-19 years were not available.

Figure S4 The ASDR of FeBGCs among AYAs. The ASDR of female breast cancer (A), cervical cancer (B), ovarian cancer (C), and uterine cancer (D) among AYAs in the globe, USA and China, from 1990 to 2019. ASDR, age-standardized disability-adjusted life year rates; AYAs, adolescents and young adults; DALY, disability-adjusted life year; FeBGCs, female breast and gynecologic cancers; USA, the United States of America. *Data on disability-adjusted life year of uterine cancer of 15-19 years were not available.

Figure S5 The rates of DALYs and deaths attributable to factors for FeBGCs among AYAs in 1990-2019. (A) Rates of DALYs (a) and deaths (b) attributable to diet high in red meat for breast cancer; (B) Rates of DALYs (a) and deaths (b) attributable to secondhand smoke for breast cancer; (C) Rates of DALYs (a) and deaths (b) attributable to high fasting plasma for ovarian cancer; AYAs, adolescents and young adults; DALY, disability-adjusted life year; FeBGCs, female breast, and gynecologic cancers; SDI, social-demographic index.

Figure S6 Time series plots of observed and predictive values of incidence and mortality rates. Observed and predictive values of incidence and mortality rates of female breast cancer (A, E), cervical cancer (B, F), ovarian cancer (C, G), and uterine cancer (D, H) incidence rates from 1990 to 2030 in China. CI, confidence interval.

Figure S7 Plots of fitting process of the ARIMA models of incidence and mortality rates. Unstable time series before differential transformations (a), ACF and PACF before differential transformations (b), stable time series plots after differential transformations (c), ACF and PACF after differential transformations (d), and residuals after differential transformations (e) of the ARIMA models of incidence rate (A) and mortality rate (B). ACF, autocorrelation coefficient; ARIMA, autoregressive integrated moving average; PACF, partial autocorrelation coefficient.

Figure S8 Plots of fitting process of the ARIMA models of incidence. Unstable time series before differential transformations (a), ACF and PACF before differential transformations (b), stable time series plots after differential transformations (c), ACF and PACF after differential transformations (d) and residuals after differential transformations (e) of the ARIMA models of breast cancer (A), cervical cancer (B), ovarian cancer (C) and uterine cancer (D). ACF, autocorrelation coefficient; ARIMA, autoregressive integrated moving average; PACF, partial autocorrelation coefficient.

Figure S9 Plots of fitting process of the ARIMA models of mortality. Unstable time series before differential transformations (a), ACF and PACF before differential transformations (b), stable time series plots after differential transformations (c), ACF and PACF after differential transformations (d), and residuals after differential transformations (e) of the ARIMA models of breast cancer (A), cervical cancer (B), ovarian cancer (C) and uterine cancer (D). ACF, autocorrelation coefficient; ARIMA, autoregressive integrated moving average; PACF, partial autocorrelation coefficient.

Table S1 Prediction of incidence and mortality of FeBGCs among AYAs in China in 2020-2030.

Table S2 Goodness of fitting tests of the ARIMA models of incidence and mortality rates.

Table S3 Goodness of fitting tests of the ARIMA models of incidence of FeBGCs.

Table S4 Goodness of fitting tests of the ARIMA models of mortality of FeBGCs.
