# Supplementary figures and images for: Epidemiological trends of female breast and gynecologic cancers in adolescents and young adults in China from 1990 to 2019: Results from the Global Burden of Disease Study 2019
Source: Front Oncol. 2022 Oct 13;12:1003710. doi: 10.3389/fonc.2022.1003710 (PMC9606349; doi:10.3389/fonc.2022.1003710)

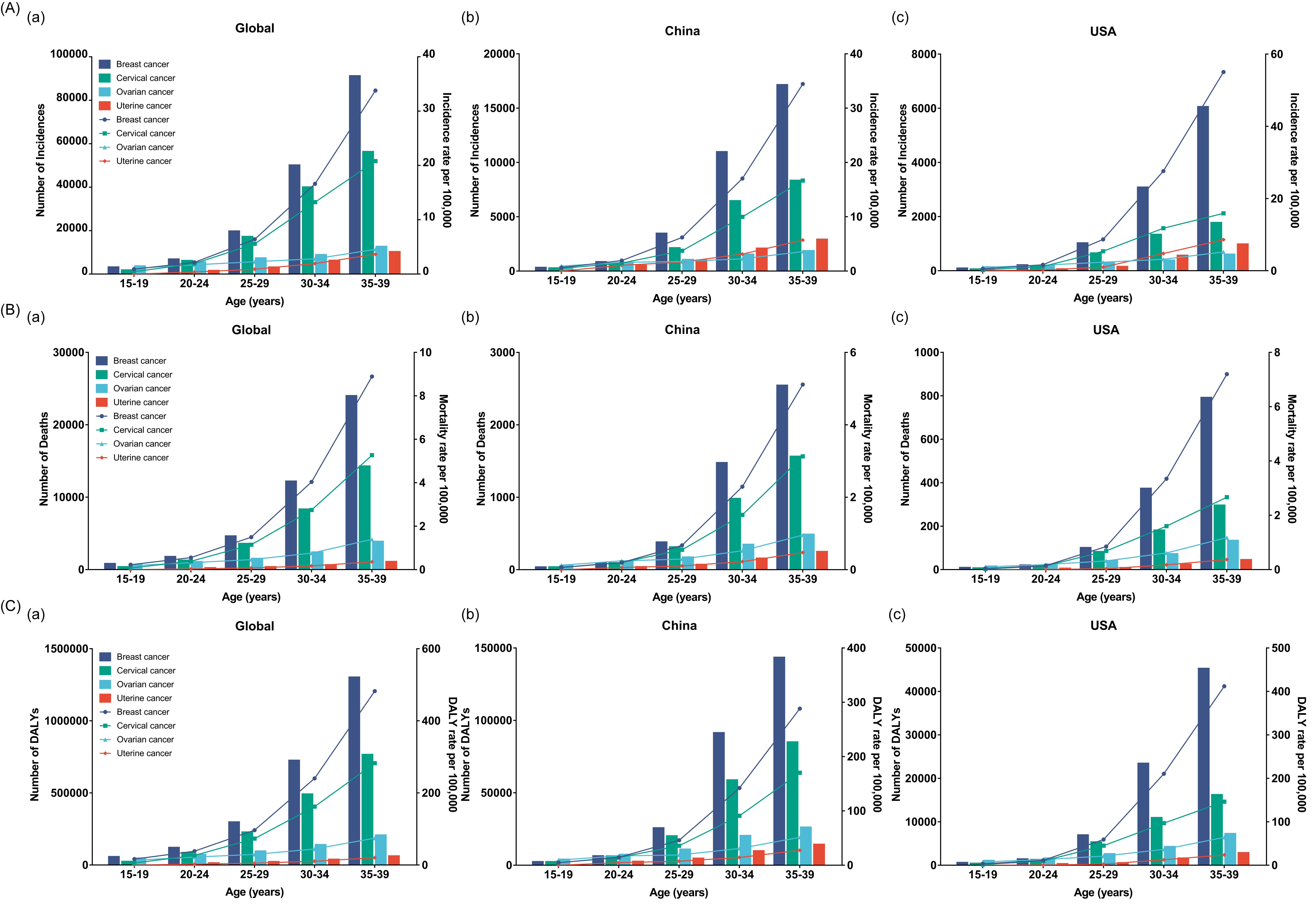

Supplement: Figure S1 — Age-specific numbers and rates of incidence, death, and DALY of FeBGCs among AYAs. Age-specific numbers and rates of Incidence (A), Death (B), and DALY (C) of female breast and gynecologic cancers among AYAs in the globe (a), China(b), and USA (c) in 2019. AYAs, adolescents and young adults; DALY, disability-adjusted life year; FeBGCs, female breast, and gynecologic cancers; USA, the United States of America. [file Image_1.tif]

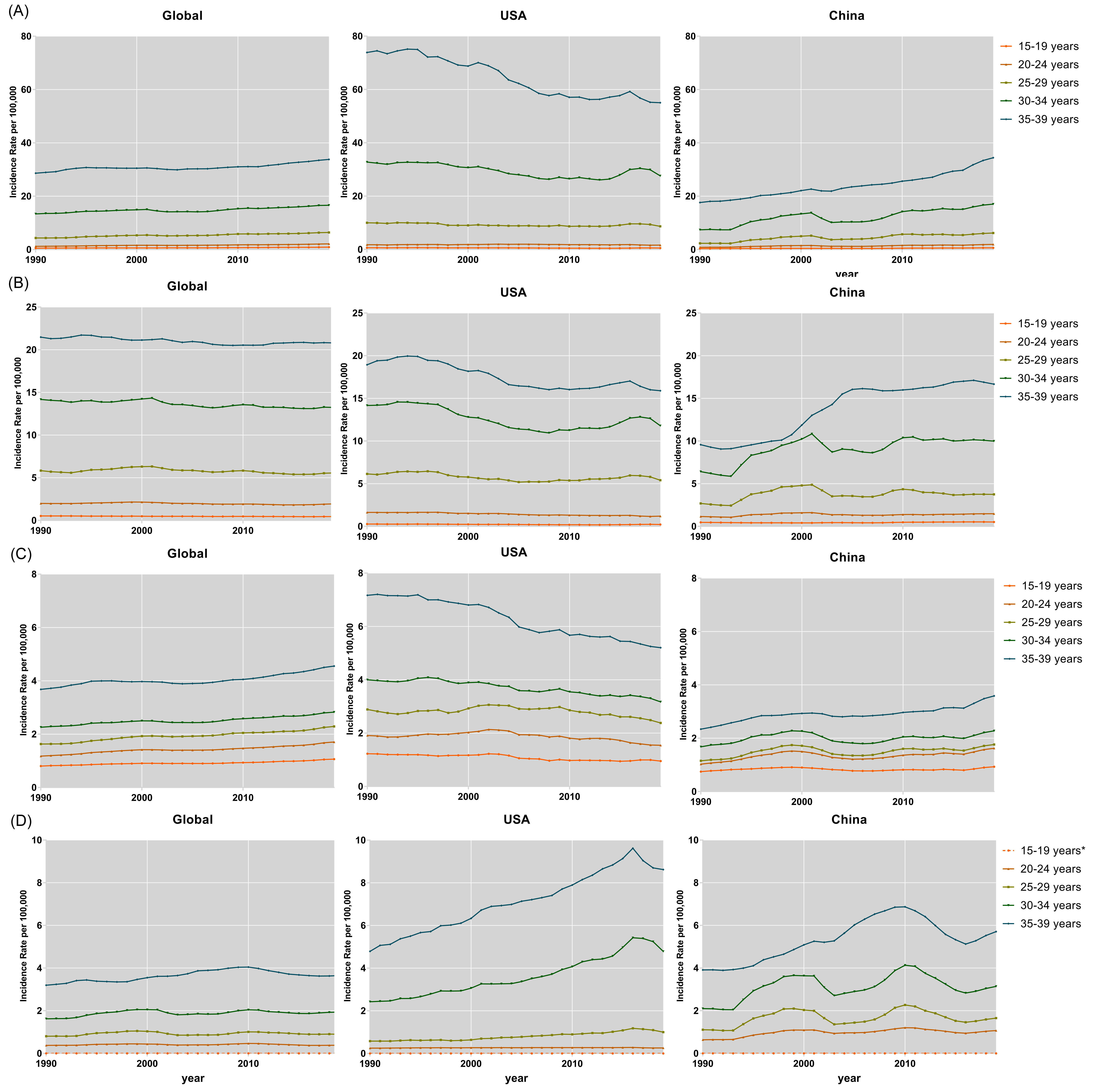

Supplement: Figure S2 — The ASIR of FeBGCs among AYAs. The ASIR of female breast cancer (A), cervical cancer (B), ovarian cancer (C), and uterine cancer (D) among AYAs in the globe, USA, and China, from 1990 to 2019. ASIR, age-standardized incidence rates; AYAs, adolescents and young adults; FeBGCs, female breast and gynecologic cancers; USA, the United States of America. *Data on incidence of uterine cancer of 15-19 years were not available. [file Image_2.tif]

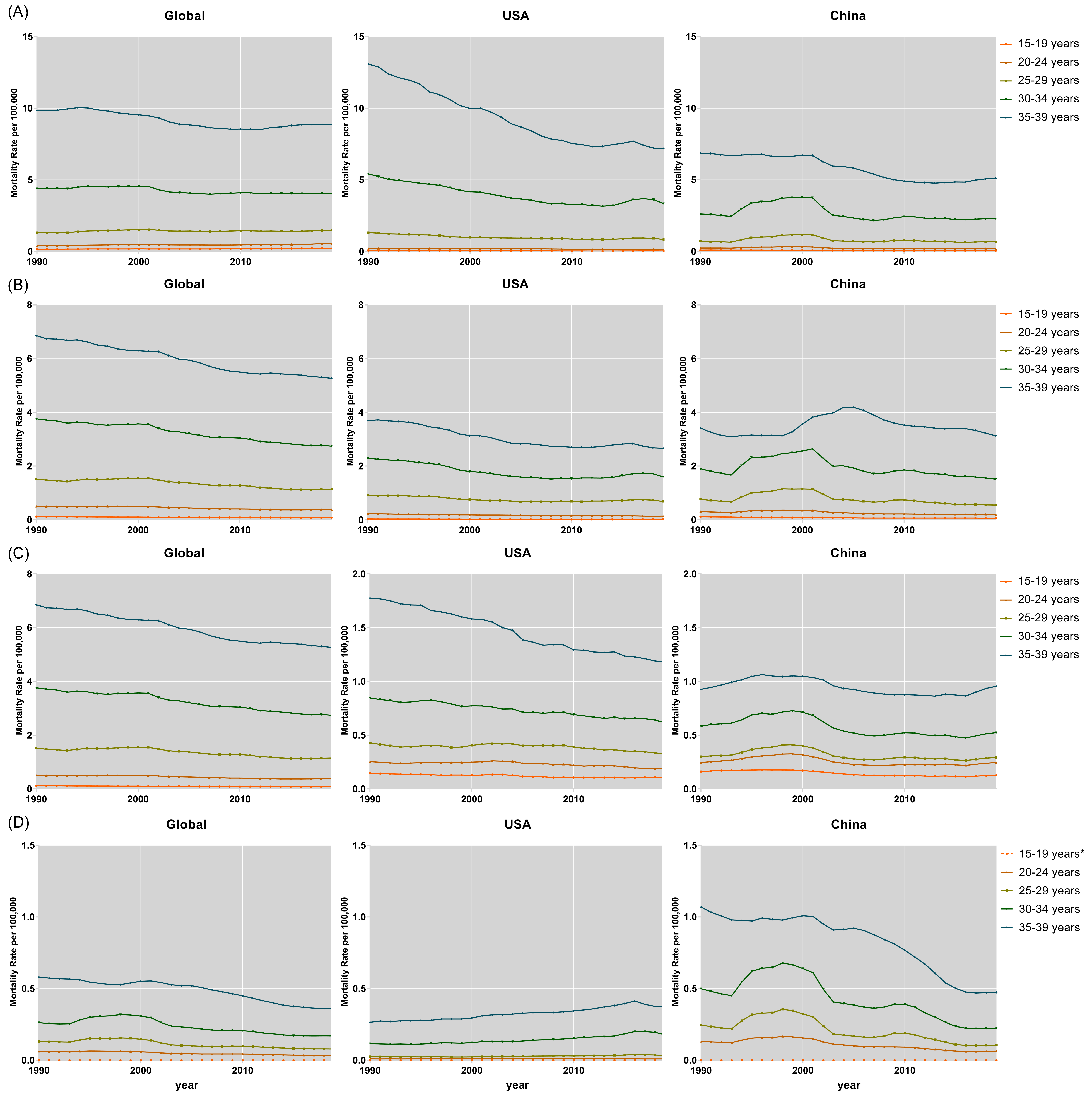

Supplement: Figure S3 — The ASMR of FeBGCs among AYAs. The ASMR of female breast cancer (A), cervical cancer (B), ovarian cancer (C), and uterine cancer (D) among AYAs in the globe, USA, and China, from 1990 to 2019. ASMR, age-standardized mortality rates; AYAs, adolescents and young adults; FeBGCs, female breast and gynecologic cancers; USA, the United States of America. *Data on mortality of uterine cancer of 15-19 years were not available. [file Image_3.tif]

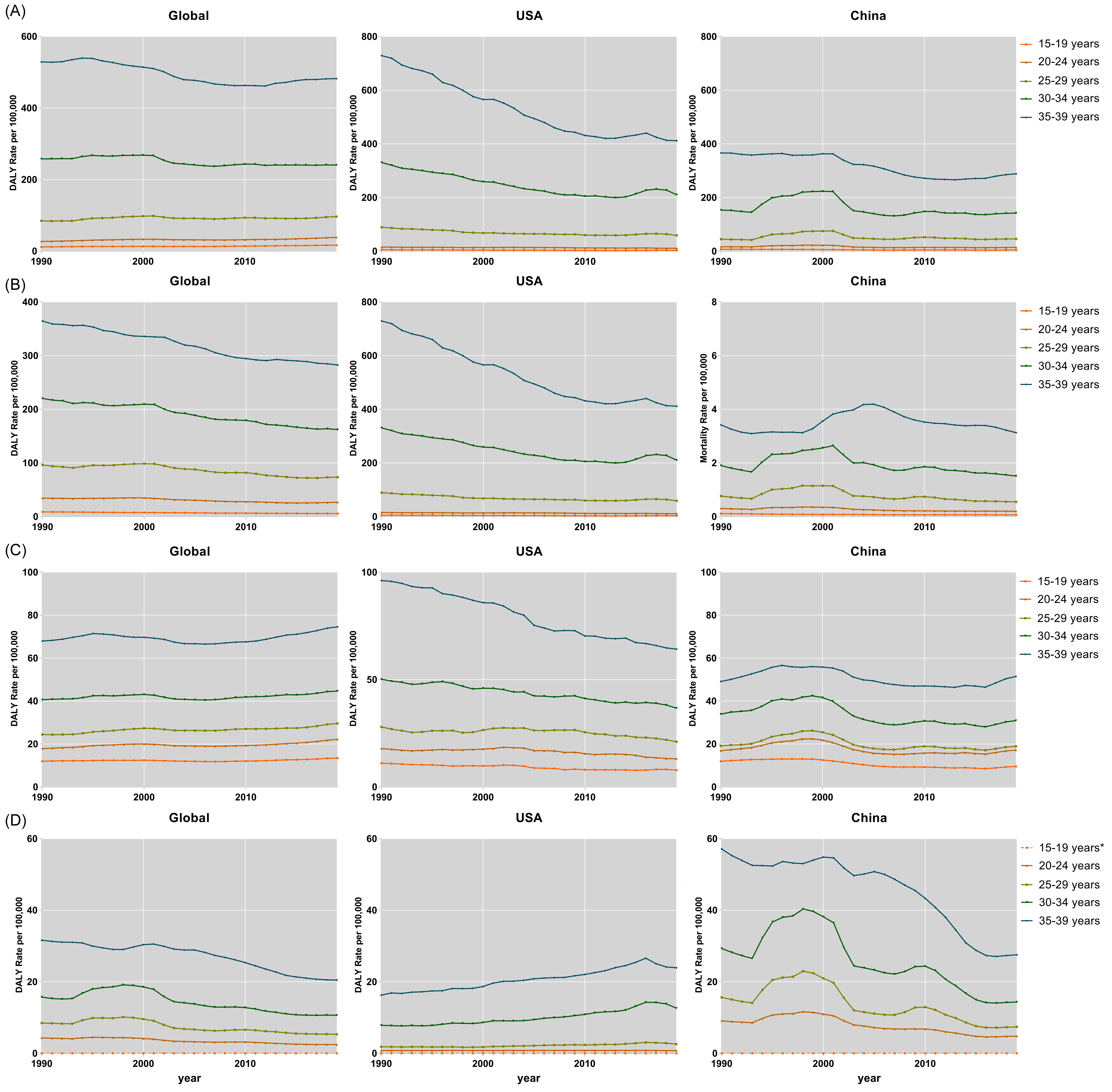

Supplement: Figure S4 — The ASDR of FeBGCs among AYAs. The ASDR of female breast cancer (A), cervical cancer (B), ovarian cancer (C), and uterine cancer (D) among AYAs in the globe, USA and China, from 1990 to 2019. ASDR, age-standardized disability-adjusted life year rates; AYAs, adolescents and young adults; DALY, disability-adjusted life year; FeBGCs, female breast and gynecologic cancers; USA, the United States of America. *Data on disability-adjusted life year of uterine cancer of 15-19 years were not available. [file Image_4.tif]

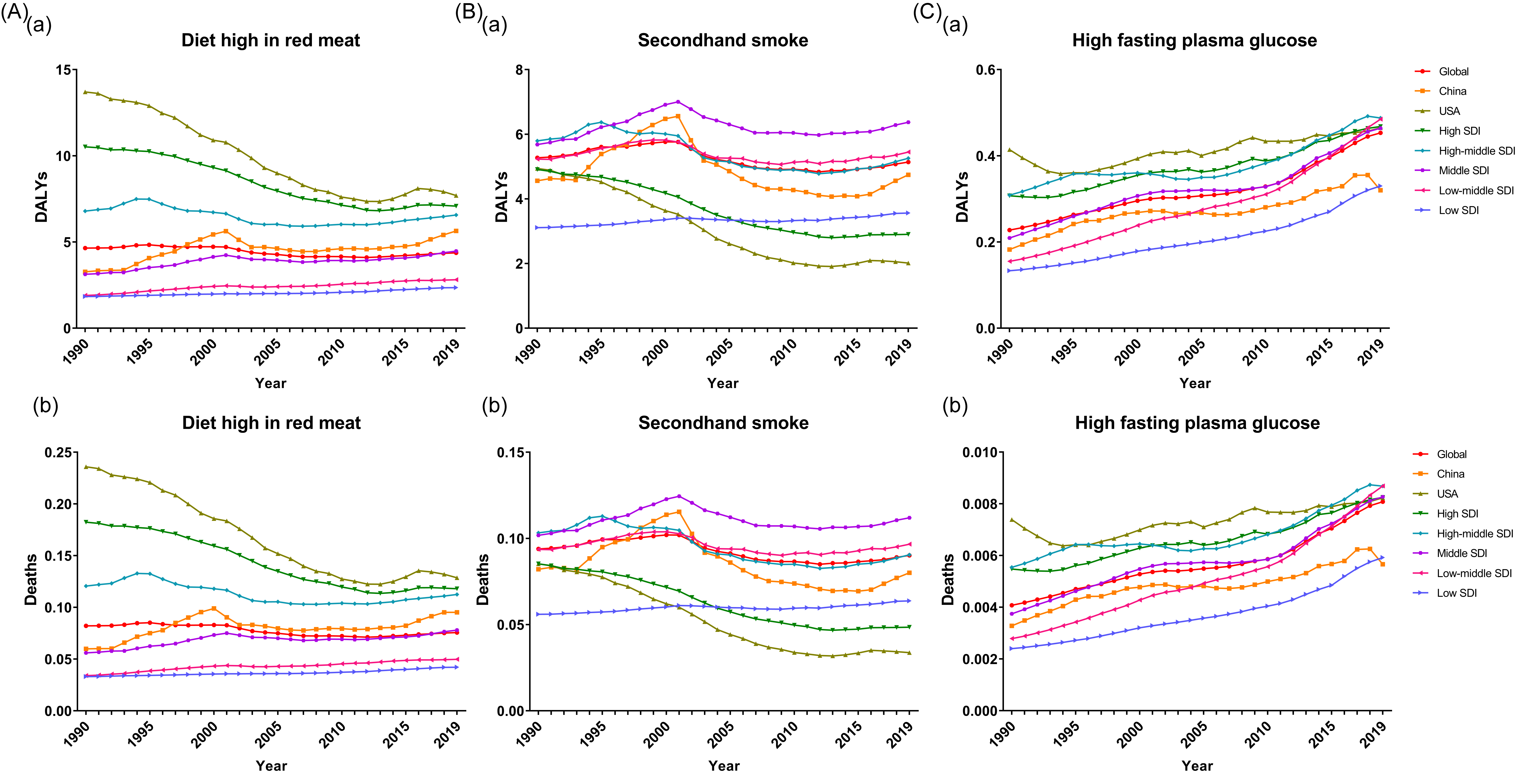

Supplement: Figure S5 — The rates of DALYs and deaths attributable to factors for FeBGCs among AYAs in 1990-2019. (A) Rates of DALYs (a) and deaths (b) attributable to diet high in red meat for breast cancer; (B) Rates of DALYs (a) and deaths (b) attributable to secondhand smoke for breast cancer; (C) Rates of DALYs (a) and deaths (b) attributable to high fasting plasma for ovarian cancer; AYAs, adolescents and young adults; DALY, disability-adjusted life year; FeBGCs, female breast, and gynecologic cancers; SDI, social-demographic index. [file Image_5.tif]

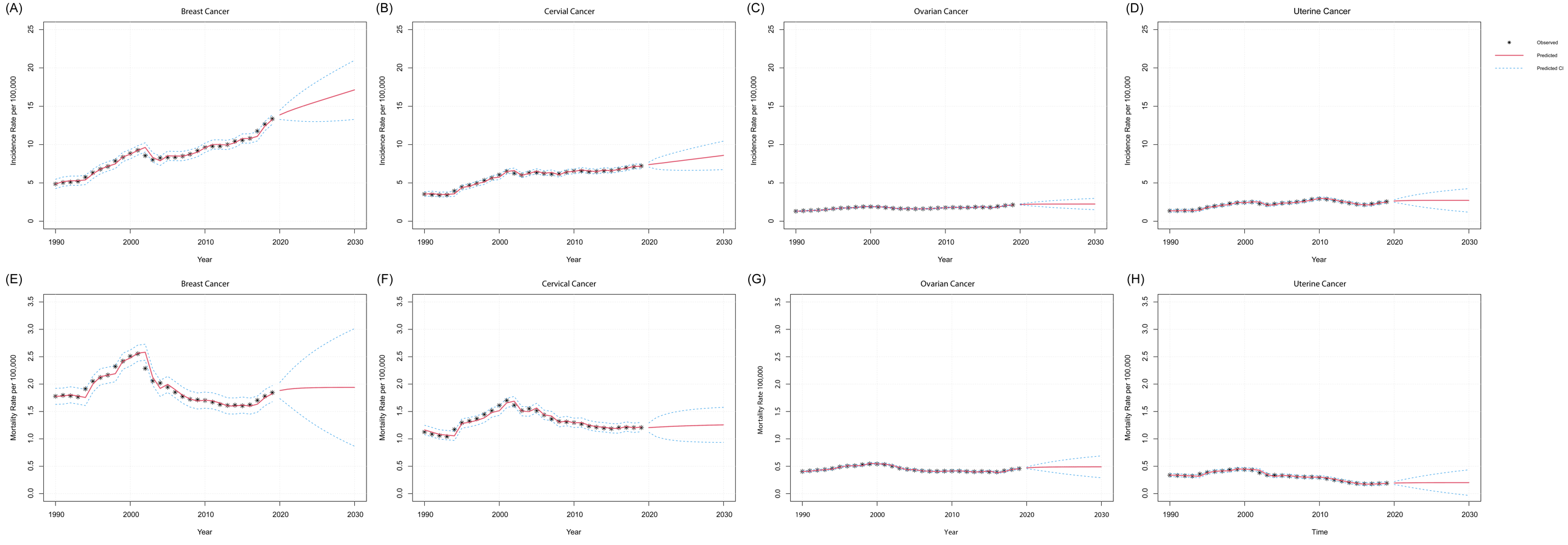

Supplement: Figure S6 — Time series plots of observed and predictive values of incidence and mortality rates. Observed and predictive values of incidence and mortality rates of female breast cancer (A, E), cervical cancer (B, F), ovarian cancer (C, G), and uterine cancer (D, H) incidence rates from 1990 to 2030 in China. CI, confidence interval. [file Image_6.tif]

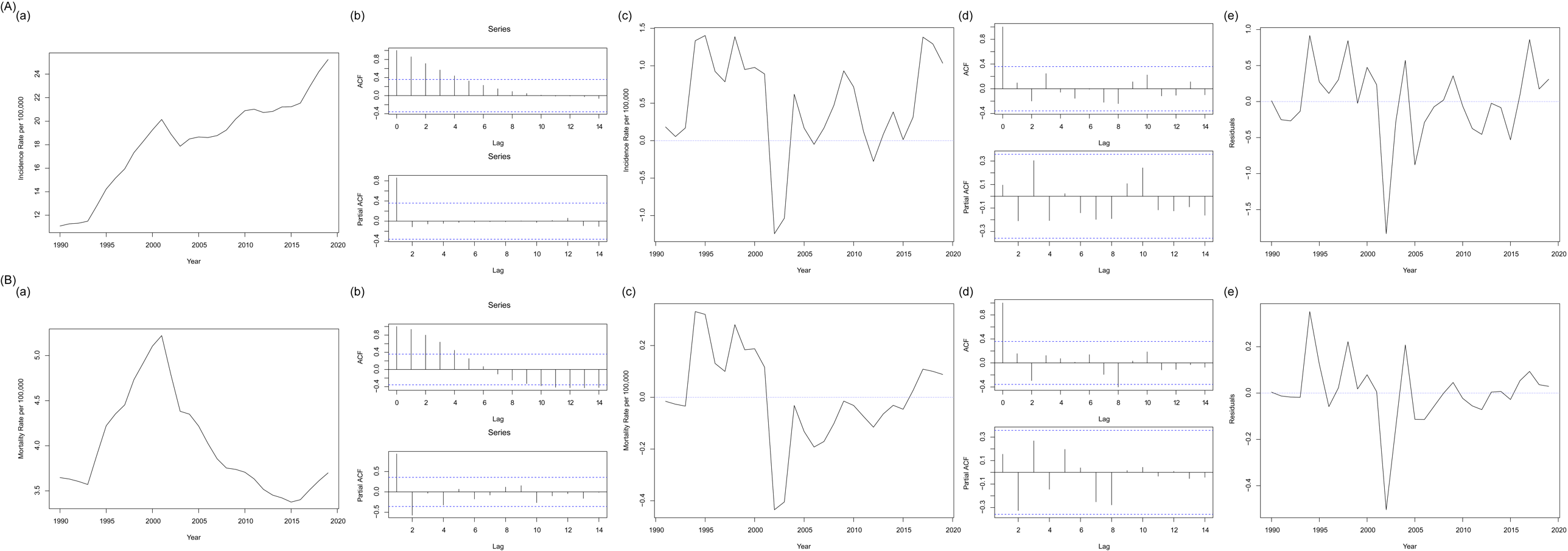

Supplement: Figure S7 — Plots of fitting process of the ARIMA models of incidence and mortality rates. Unstable time series before differential transformations (a), ACF and PACF before differential transformations (b), stable time series plots after differential transformations (c), ACF and PACF after differential transformations (d), and residuals after differential transformations (e) of the ARIMA models of incidence rate (A) and mortality rate (B). ACF, autocorrelation coefficient; ARIMA, autoregressive integrated moving average; PACF, partial autocorrelation coefficient. [file Image_7.tif]
